# Energy collaborative optimization of power routing based on PPO and generative adversarial imitation learning

**DOI:** 10.1371/journal.pone.0346372

**Published:** 2026-04-08

**Authors:** Junyan Lyu, Jing Huang

**Affiliations:** School of Electronic, Electrical Engineering and Physics, Fujian University of Technology, Fuzhou, China; Wuhan University, CHINA

## Abstract

Under the general trend of global energy transformation, the proportion of renewable energy in the power sector continues to increase. Power routers are of great significance for improving energy utilization efficiency and ensuring the stable operation of power systems. However, the intermittent and uncertain nature of distributed energy makes energy management of power routers difficult, and traditional optimization methods are also difficult to adapt. Therefore, this study proposes the integration of Proximal Policy Optimization with a multi-agent framework, combined with a Generative Adversarial Imitation Learning based on a double-buffer mechanism. The double-buffer mechanism is used to improve data utilization efficiency and training stability, and to optimize communication and collaboration among multiple agents, thereby realizing energy collaborative optimization of power routers. Experimental results show that after 420 trainings, the average round reward of the improved algorithm is stable at about −410, and the strategy loss function is the first to stabilize after 500 times. In practical scenarios, the proposed model maintains a DC bus voltage fluctuation range between 728V and 732V. Additionally, its electricity cost amounts to 3846.36 yuan, and its total runtime is 53.32 seconds—both of which are lower than those of the other two models. Overall, the enhanced algorithm and model notably improve the energy collaboration optimization of power routers, offering a practical solution to energy management issues and significantly advancing the progress in this area.

## 1. Introduction

With the global drive for energy transition, the proportion of renewable energy in the power sector is steadily increasing. Power routers are key devices in this field, and their energy collaborative optimization is vital for improving energy utilization efficiency and ensuring the stable operation of power systems [1]. However, the inherent intermittency and uncertainty of distributed energy introduce numerous challenges in energy management for power routers [2]. Traditional methods, when faced with the complex energy management scenarios of power routers, expose limitations in adaptability [3]. Therefore, exploring more efficient and precise energy collaborative optimization strategies has become a core research direction in this field. Generative Adversarial Imitation Learning (GAIL) leverages the adversarial mechanism of a generator and a discriminator to allow agents to learn expert policies, effectively avoiding the difficulties in designing complex reward functions [4]. Scholars worldwide have achieved significant results in related studies. For example, the Bhattacharyya team proposed an improved GAIL model to address the challenge of building reliable human driving behavior models for autonomous vehicle simulation, framing driving as a sequential decision-making problem. Experiments show that this method can simulate high-speed driving behavior [5]. Proximal Policy Optimization (PPO) enhances the stability and efficiency of the policy optimization process by effectively controlling the step size of policy updates [6]. Scholars such as Kuai et al. proposed a deep reinforcement learning method based on offline PPO to address the integer constraints in network function virtualization mapping and scheduling, with experiments showing its superior performance in terms of service fairness and acceptance rate compared to traditional algorithms [7]. Research on energy collaborative optimization has also been actively carried out. For example, the Chen team proposed a multi-drone collaborative scheme with sensing task overlapping assignment to fully exploit the advantages of multi-drone sensing and communication, deriving a solution to the sensing condition transformation problem. Experiments demonstrate the superiority of their approach [8]. Ferro et al. proposed a new system architecture and distributed optimization algorithm for the interconnected energy hubs in smart city energy communities, ensuring information privacy while accelerating iterative solutions. Evaluation results indicate that their method outperforms others in terms of savings, convergence, and scalability [9]. Hashemipour’s team proposed a probabilistic modeling approach to address the uncertainty of electric vehicles in peer-to-peer transactions. This method does not rely on the shape of the probability density function but captures uncertainty using random vectors and fuzzy membership functions. Experiments show that this method is more accurate and computationally efficient, effectively handling related time uncertainties [10]. In order to identify system state changes in the multi scene edge cloud native environment, Xiong et al. Designed a multi-agent Gail method for training in the virtual environment. It introduced a virtual environment, which can generate the interaction of multi scene mixed service groups. The results show that the performance is significantly better than the traditional supervised learning method [11]. For the energy management of electric vehicle batteries, Xu’s team proposed a safety reinforcement learning method based on power allocation of electric vehicle hybrid energy storage system based on PPO and predictive safety filter. It combines reinforcement learning and predictive safety filter, and designs incentive rewards to guide the training process of reinforcement learning. The results show that the training time is significantly reduced compared with the traditional PPO method [12].

Despite previous research on improving algorithms for energy management in power routers, there remains significant room for exploration in this field. The existing algorithms have significant limitations when dealing with the uncertainties of distributed energy. When traditional GAIL imitates expert strategies, it uses a single buffer to store data, making it difficult to distinguish the priority of expert data from the interaction data of the generator, resulting in low sample utilization and prone to policy oscillations during the training process. In multi-agent scenarios, the traditional PPO algorithm lacks effective communication and attention mechanisms among agents, resulting in low information processing efficiency. In a dynamic energy environment, the existing multi-agent reinforcement learning methods rely on global experience playback for policy updates, ignoring the local characteristics of each agent, which leads to an increase in the operating cost of the system. Therefore, this study proposes a GAIL algorithm based on a double buffering mechanism (DB-GAIL). Compared to traditional methods, the dual-buffered separation architecture of the proposed DB-GAIL fundamentally solves the problem of expert high-value samples being mixed with generator exploration data in single-buffered GAIL, leading to the dilution of expert policies. It also avoids the limitations of PER-based methods, which rely solely on temporal difference (TD) error allocation for priority assignment and cannot adapt to the dual-data characteristics of imitation learning. The hybrid priority sampling strategy proportionally merges expert samples and generator high-value exploration samples, ensuring effective transfer of expert policies while enhancing the utilization efficiency of high-information-gain samples. Compared to single-buffered uniform sampling and PER’s single-priority sampling, it significantly improves sample utilization. Simultaneously, the dual mechanisms work together to reduce policy update variance during training, addressing the issues of drastic training fluctuations in single-buffered GAIL and policy shifts in PER-based methods due to the lack of data stratification. This improves the utilization of high-value samples and reduces training fluctuations. Furthermore, considering the problem of low communication and collaboration efficiency among agents in the multi-agent scenario of the traditional PPO algorithm, this study proposes a multi-agent PPO algorithm (MAPPO). By introducing an attention mechanism to focus on the status of key devices, it optimizes the communication and collaboration mechanisms among agents, shortens the delay of collaborative decision-making, and controls the deviation rate of energy distribution. This will enhance the collaborative efficiency of energy management in power routers. The study explores the application of the improved MAPPO and DB-GAIL algorithms for energy collaborative optimization in power routers and incorporates Deep Deterministic Policy Gradient with Prioritized Experience Replay (DDPG-PER) to further enhance the system model. The study innovatively proposes DB-GAIL and MAPPO for collaborative optimization. DB-GAIL improves data utilization efficiency and training stability, while MAPPO optimizes communication and collaboration among multiple agents. The aim is to provide a new approach for energy management in power routers, effectively addressing the uncertainty of distributed energy and enhancing overall system performance.

## 2. Methods and materials

### 2.1. Integration strategy of improved gail and ppo

Energy routers play an important role in integrating distributed energy resources and improving electricity utilization efficiency. However, the intermittency and uncertainty of distributed energy bring challenges to the energy collaborative optimization of energy routers [13]. Traditional energy management methods are inadequate to handle complex and dynamic operational scenarios [14]. GAIL has unique advantages in imitating expert strategies and acquiring effective strategies [15]. PPO limits the update range of new and old policies with a clipping method, balancing stability and simplicity. Its architecture includes an actor and a critic network. The commentator network assesses the expected value and calculates the dominance function, which quantifies the additional expected benefits that can be brought by choosing a specific action in a specific state compared to the average action following the current strategy. Its expression is shown in Formula (1) [16].


$$A_{\pi }\left(s,a\right)=Q_{\pi }\left(s,a\right)-V_{\pi }\left(s\right)$$ (1)

In Equation (1), s represents the state, a indicates the action, and π is the policy, determining the mapping from state to action. Q is the action value function, used to evaluate the expected future cumulative reward after executing the action in a given state under the policy. V is the state value function. When the critic network performs state estimation, the Monte Carlo method estimates the state value function,. The Monte Carlo method fully simulates a round starting from state s(t) until the end time T, collects the actual cumulative discount reward $G_{t}$ starting from s(t) in this round, and uses it as an estimate of $V_{M}\left(s\left(t\right)\right)$. Then, use this estimate to update the approximate value of $V_{M}\left(s\left(t\right)\right)$. The calculation is as shown in Equation (2) [17].


$$\left\{ \begin{array}{c} @l@\overset{\;\prime }{\underset{M}{V}}(s(t))=V_{M}(s(t))+a(G(t)-V_{M}(s(t))\\ G_{t}=r(t+1)+\gamma r(t+2)+\gamma^{\text{2}}r(t+3)+\cdot \cdot \cdot +\gamma^{T-t-1}r(T) \end{array}\right.$$ (2)

In Equation (2), s(t) represents the state and environment information at time t. $V_{M}\left(s\left(t\right)\right)$ is the state value of s(t) at time t, while $\overset{\text{'}}{\underset{M}{V}}\left(s\left(t\right)\right)$ updates the state value function. a controls the update step size with the learning rate. $G_{t}$ is the accumulated future reward, r(t+1) is the reward obtained by the agent, γ is the discount factor, and T is the termination time. The Monte Carlo method operates without the need for prior knowledge of the environment, whereas temporal difference learning merges its benefits with dynamic programming, updating based on the temporal difference error [18]. The evaluation network optimizes its parameters through a specific loss function and gradient descent. The parameters of the evaluation network are optimized by minimizing a loss function and using gradient descent. This loss function measures the difference between the state value predicted by the commentator network and a certain target value. The calculation method is shown in Equation (3).


$$\left\{ \begin{array}{c} @l@L_{c}(\varphi )=\frac{\text{1}}{N}\sum_{i=1}^{N}(R(i)-V(s(i)){)}^{\text{2}}\\ \varphi^{\prime }=\varphi -\alpha \nabla_{\varphi }L_{c}\left(\varphi \right) \end{array}\right.$$ (3)

In Equation (3), φ represents the parameters of the actor network. N is the number of samples, R(i) is the actual reward of the i -th sample, $\varphi^{\text{'}}$ is the updated parameter set, and $\nabla_{\varphi }L_{c}\left(\varphi \right)$ is the gradient of the loss function with respect to parameter φ. A clipping function is introduced to limit the advantage function, correcting and optimizing the target function with gradient ascent, as shown in Equation (4).


$$\left\{ \begin{array}{c} @l@L_{clip}(\omega )=E_{t}[min(u_{t}(\omega )A(t),clip(u_{t}(\omega ),1-\varepsilon ,1+\varepsilon )A(t)]\\ u_{t}\left(\omega \right)=\frac{\pi \omega (a(t)|s(t))}{\pi \omega_{old}(a(t)|s(t))}\\ \omega^{\prime }=\omega +\beta \nabla_{\omega }L\left(\omega \right) \end{array}\right.$$ (4)

**Fig 1:**
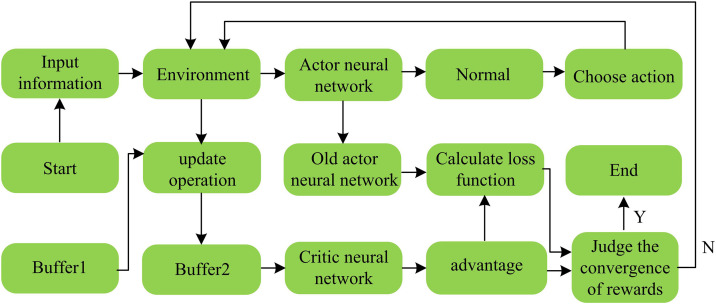
Schematic diagram of PPO operation process.

In Equation (4), E represents the expected value, min is the minimum operation, $u_{t}\left(\omega \right)$ is the probability ratio between the new policy network πω(a(t)|s(t)) and the old policy network $\pi \omega_{old}(a(t)|s\left(t\right))$, A(t) is the advantage function, clip is the clipping function, ω is the actor network parameter, β is the learning rate, and ε is a preset hyperparameter. The PPO flowchart is shown in Fig 1.

As shown in Fig 1, the process starts with obtaining input information, then interacting with the environment. The generated raw data such as state, action, and reward are stored in buffer 1, which serves as a temporary storage for raw information. After the environment is updated, the relevant data enters buffer 2 for subsequent key calculations. The data in buffer 2 is input into the policy neural network, which selects actions after normal distribution processing, acts on the environment and records information. At the same time, its data is used to calculate the advantage function of the value neural network. The old parameters of the policy neural network are combined with the advantage function to calculate the loss. The advantage function is also used to judge the convergence of the reward. If the convergence process ends, if it does not converge, it returns to continue iterating. In the energy collaborative optimization of power routers, single-agent PPO is difficult to handle multi-device interactions. MAPPO, as a multi-agent extension of PPO, allows agents to optimize strategies based on local and global information, and can also combine generative adversarial imitation learning to achieve efficient collaboration. The definition of the Actor-Critic network update of MAPPO is shown in Equation (5) [19].


$$\left\{ \begin{array}{c} @l@\theta^{\prime }=\theta +\beta \nabla_{\theta }E_{t}[min(u_{t}(\theta )A(t),clip(u_{t}(\theta ),1-\varepsilon ,1+\varepsilon )A(t)]\\ \phi^{\prime }=\phi -\alpha \nabla_{\phi }\frac{\text{1}}{N}\sum_{t=1}^{N}[R(t)-V_{\phi }(s(t)){]}^{\text{2}} \end{array}\right.$$ (5)

**Fig 2:**
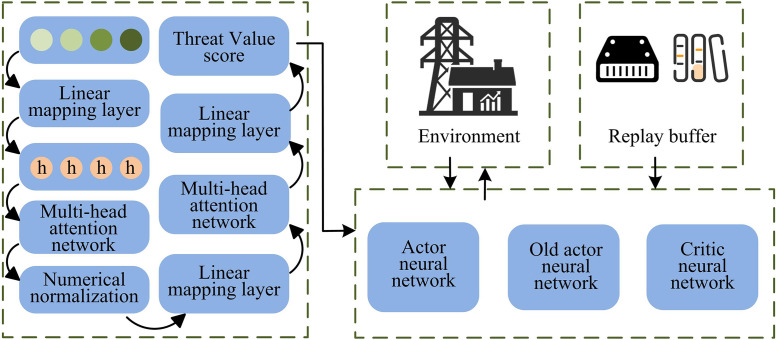
Schematic diagram of the ATT-MAPPO framework.

In Equation (5), θ represents the parameters of the agent’s actor network, $\theta^{\text{'}}$ indicates the updated parameters, while ϕ and $\phi^{\text{'}}$ represent the pre- and post-update parameters of the critic network. Although MAPPO resolves multi-agent policy optimization problems to some extent, it still has shortcomings in handling complex and dynamic power environments, such as inefficient and inaccurate information processing and difficulty in focusing on key tasks [20]. The Attention Mechanism (ATT) allows the model to focus on critical parts of the information during processing, improving the accuracy and efficiency of information handling. Therefore, we introduce the ATT mechanism to improve MAPPO (ATT-MAPPO), which better meets the energy optimization needs of energy routers. The ATT-MAPPO framework is shown in Fig 2.

In Fig 2, the process first processes information, then normalizes the input data for preprocessing, and the multi-head attention network captures key information, which is then processed by the linear mapping layer to finally obtain the threat value score. The middle environment module interacts with the agent, and the storage unit on the right acts as an experience temporary pool, responsible for saving various types of experience data records generated. The strategy and value evaluation module consists of the policy neural network, the old policy neural network, and the value neural network. The policy neural network generates action strategies based on environmental data, the old policy neural network stores previous parameters, and the value neural network assesses the value of each state. ATT-MAPPO improves the processing ability of key information through the attention mechanism, optimizes the collaboration and decision-making among multiple agents, but relying solely on its own exploration learning in complex environments may be inefficient. ATT-MAPPO enhances the processing capability of critical information and optimizes collaboration and decision-making among multiple agents through an attention mechanism. In multi-agent energy management scenarios, the weight of each device’s state on the overall system dynamically changes. The attention mechanism can adaptively identify and focus on the state variables most critical to system stability at the current moment. Power router systems contain a large amount of redundant state information; the attention mechanism effectively filters noise and improves decision-making efficiency by calculating the correlation between states. In multi-agent collaboration, the attention mechanism can model the dependencies between agents, achieving more efficient collaborative decision-making. In experimental environments, state variables such as DC bus voltage deviation, energy storage device state of charge (SOC), renewable energy output volatility, and critical load demand significantly benefit from the attention mechanism. These state variables are typically treated equally in traditional methods, while the attention mechanism dynamically adjusts its focus based on the current system state, thereby improving decision quality. GAIL mainly uses adversarial training between the generator network and the discriminator network to allow the agent to learn expert strategies [21,22]. The discriminator network takes the state-action pair as input, and its loss function is formulated as shown in Equation (6).


$$\left\{ \begin{array}{c} @l@L\left(\vartheta \right)=-E_{\rho_{\varepsilon }}\left[log\;D_{\vartheta }\left(s_{t},a_{t}\right)\right]-E_{\rho_{E}}\left[log\left(1-D_{\vartheta }\left(s_{t},a_{t}\right)\right)\right]\\ \vartheta =\vartheta -\sigma_{D}\nabla_{\vartheta }L\left(\vartheta \right) \end{array}\right.$$ (6)

In Equation (6), ϑ represents the discriminator network’s parameters, $E_{\rho_{\varepsilon }}$ is the expectation over the data generated by the generator, $E_{\rho_{E}}$ is the expectation over expert data, $D_{\vartheta }\left(s_{t},a_{t}\right)$ is the discriminator’s output, $\sigma_{D}$ is the discriminator’s learning rate, and $\nabla_{\vartheta }L\left(\vartheta \right)$ is the gradient of the loss function with parameter ϑ, indicating the update direction for the parameters. The reward function for the generator network is defined as shown in Equation (7).


$$r\left(s_{t},a_{t}\right)=log\;D_{\vartheta }\left(s_{t},a_{t}\right)$$ (7)

**Fig 3:**
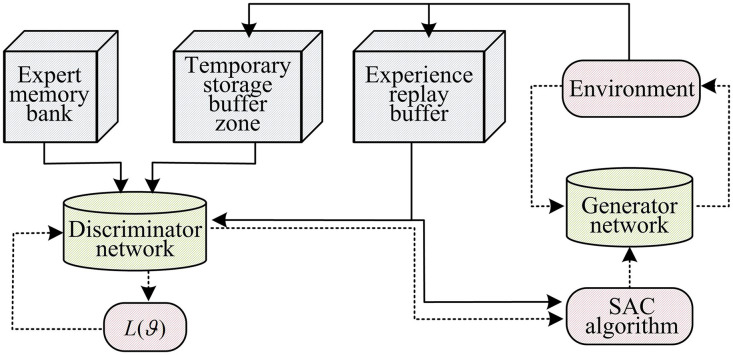
Schematic diagram of DB-GAIL framework.

In Equation (7), $r\left(s_{t},a_{t}\right)$ represents the reward function value for the generator network. Traditional GAIL suffers from low sample efficiency and limited training performance. To address this, we introduce the Dual-Buffer (DB) mechanism, which stores both expert data and data generated during the generator network’s training process, accumulating learning experience [23]. The DB mechanism consists of an expert data buffer and a generator interaction buffer. The two achieve the orderly flow and utilization of data through the coordination and scheduling module. Specifically, the expert data buffer stores samples of state-action pairs generated by high-quality expert policies. This buffer adopts an incremental update + sliding window mechanism, that is, when new expert samples are generated, they are appended in chronological order and the earliest expired samples are removed simultaneously. The generator interaction buffer stores in real time the state-action-reward-next state samples generated during the interaction between the generator network and the environment. This buffer adopts a priority update mechanism. Different priorities are assigned to samples based on their temporal difference errors. Samples with larger temporal difference errors are given higher priorities and have a higher probability of being sampled during training. The implementation of the DB mechanism involves four steps. Firstly, data collection is carried out. The expert strategy generates high-quality samples through interaction with the environment and continuously stores them in the expert data buffer. The generator network interacts with the environment in real time during the training process, and the generated exploratory samples are stored in the generator interaction buffer according to priority. Then, data sampling is carried out. During each training iteration, the coordination and scheduling module randomly samples 20% of the expert samples from the expert data buffer and 80% of the generator samples from the generator interaction buffer according to priority. The two types of samples are mixed to form the training batch. Then, data updates are carried out. The expert data buffer is updated every 100 iterations, removing expired samples and supplementing with new expert samples. The generator interaction buffer is updated immediately after each step of interaction, and the sample priority is adjusted based on the newly calculated time difference error to achieve dynamic optimization. Finally, the discriminator and the generator are trained. Mixed samples are input into the discriminator network to optimize its ability to distinguish between expert data and generator data. Meanwhile, the rewarded samples stored in the generator interaction buffer are input into the generator network. Combined with the policy evaluation output by the discriminator, the generator policy is optimized through the Soft Behavior Critic (SAC) algorithm to gradually approach the expert policy in the generated action sequence. With the DB mechanism, we adapt the off-policy deep reinforcement learning algorithm and utilize the SAC algorithm’s powerful policy optimization and efficient learning capabilities to continuously adjust the strategy and generate state-action pairs that approximate the expert strategy, resulting in better learning outcomes [24]. The improved DB-GAIL architecture is shown in Fig 3.

**Fig 4:**
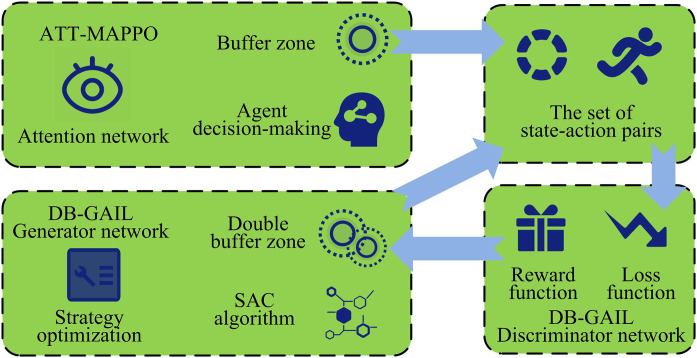
Schematic diagram of the hybrid algorithm structure.

In Fig 3, the expert memory stores expert data, the temporary storage buffer stores data generated during generator network training, and the experience replay buffer accumulates past interaction experience data. The data from these three sources are fed into the discriminator network to compute the loss function. Additionally, the data stored in the experience replay buffer is passed to the generator network, which then interacts with the environment to produce new data. At the same time, the generator network combines the output of the discriminator to construct a reward function, and continuously optimizes its own strategy with the help of the SAC algorithm to generate state-action pairs that are closer to the expert strategy. With the attention mechanism, ATT-MAPPO can effectively improve the processing ability of key information and optimize the collaboration and decision-making among multiple agents. DB-GAIL has unique advantages in learning expert strategies. The combination of the two can achieve complementary advantages. The structure diagram is shown in Fig 4.

As shown in Fig 4, the storage unit of the ATT-MAPPO part is an experience temporary pool to save various data records generated after communicating with the environment. After the attention network preprocesses the input data by numerical normalization, it captures key information and obtains the threat value score through linear mapping layer processing to assist the agent in making decisions. The policy neural network generates action strategies based on environmental information, the old policy neural network retains old parameters, and the value neural network evaluates the state value. The DB of the DB-GAIL part stores expert data and generator network training data respectively. The generator network uses the reward function constructed based on the DB data and the output of the discriminator network to optimize the strategy with the help of the SAC algorithm to generate state-action pairs close to the expert strategy. The discriminator network receives the DB data to calculate the loss function and improves the ability to distinguish data from different sources.

### 2.2. Construction of energy collaborative optimization model based on improved algorithms

**Fig 5:**
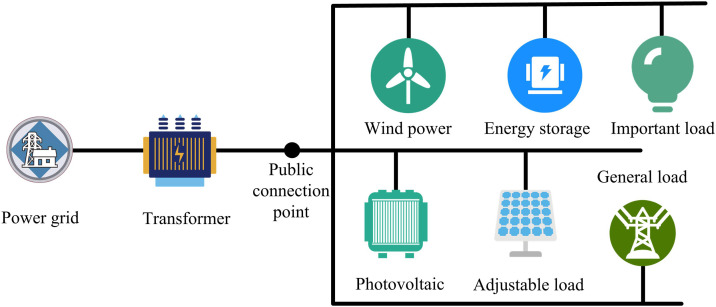
Schematic diagram of microgrid structure.

After the improvement and integration of GAIL and PPO, the study constructs an energy collaboration optimization model based on this. In the research of energy collaboration optimization for the energy router, building a microgrid model is a key step in achieving energy collaboration optimization. As a system containing various energy devices, its operating state is complex and variable [25]. The microgrid structure diagram is shown in Fig 5.

In Fig 5, the microgrid is connected to the microgrid at the public connection point through a transformer. There are various energy devices and loads in the microgrid. Wind power and photovoltaic power are constrained by natural conditions and their power generation is unstable. Energy storage is used to balance energy. Different loads have different requirements for power supply reliability. The construction of a microgrid model is of great significance to the energy management and collaborative optimization of power routers. It can clearly show the characteristics, relationships and interactions of energy devices with the outside world, and provide a basis for optimization strategies. Reasonable energy scheduling can improve utilization efficiency, reduce costs, meet electricity demand, and ensure stable and efficient operation of power routers. When constructing this model, power balance is one of the key elements, and its expression is shown in Equation (8) [26].


$$P_{load}\left(t\right)=P_{pv}\left(t\right)+P_{dg}\left(t\right)+P_{es}\left(t\right)+P_{grid}\left(t\right)$$ (8)

In Equation (8), $P_{load}$ represents the load power demand of the microgrid, $P_{pv}$ is the output power of the photovoltaic cells, $P_{dg}$ is the output power of the diesel generator, $P_{es}$ represents the charging and discharging power of the energy storage device, and $P_{grid}$ is the power exchange with the main grid. In the optimization model, the core objective is to minimize operating costs, and the objective function is shown in Equation (9).


$$min\overset{T}{\underset{t=1}{\sum }}\left[C_{pv}\left(t)+C_{dg}(t)+C_{grid}(t\right)\right]$$ (9)

In Equation (9), $C_{pv}$ represents the cost of photovoltaic generation, $C_{dg}$ is the cost of diesel generation, $C_{grid}$ is the cost of interaction with the main grid, and T is the optimization time period. Transforming the microgrid energy management problem into a Markov decision process is an important step in optimization. In the state space definition, the state vector is shown in Equation (10) [27].


$$s\left(t\right)=\left[P_{load}\left(t\right),P_{pv}\left(t\right),SOC\left(t\right),\lambda \left(t\right)\right]$$ (10)

In Equation (10), SOC is the State of Charge (SOC) of the energy storage device, usually ranging from 0 to 1, and λ represents the electricity price. The action space is defined as the set of actions that the agent can take, and its expression is shown in Equation (11).


$$P_{gen}\left(t\right)=P_{load}\left(t\right)+P_{stor}\left(t\right)+P_{grid}\left(t\right)$$ (11)

In Equation (11), $P_{gen}$ represents the total power generation of the system, $P_{load}$ and $P_{stor}$ refer to the load power and the energy storage device’s charging or discharging power, while $P_{grid}$ represents the power flow with the main grid. For the energy storage device, its SOC change follows the formula shown in Equation (12).


$$a\left(t\right)=\left[\overset{set}{\underset{dg}{P}}\left(t\right),\overset{set}{\underset{es}{P}}\left(t\right),\overset{set}{\underset{grid}{P}}\left(t\right)\right]$$ (12)

In Equation (12), $\overset{set}{\underset{dg}{P}}$ is the set diesel generator output power, $\overset{set}{\underset{es}{P}}$ is the set charging/discharging power of the energy storage device, and $\overset{set}{\underset{grid}{P}}$ is the power exchange with the main grid at a given time. The reward function is used to guide the agent in learning the optimal strategy, and its design formula is shown in Equation (13).


$$R\left(s\left(t\right),a\left(t\right)\right)=-bC_{total}\left(t\right)-\beta \left|P_{load}\left(t\right)-P_{pv}\left(t\right)-\overset{set}{\underset{dg}{P}}\left(t\right)-\overset{set}{\underset{es}{P}}\left(t\right)-\overset{set}{\underset{grid}{P}}\left(t\right)\right|=\gamma \Delta SOC^{\text{2}}\left(t\right)$$ (13)

In Equation (13), $C_{total}$ represents the total operating cost. b, β, and γ are the weight coefficients. ΔSOC represents the change in the SOC of the energy storage device. This term is introduced to penalize excessive changes in the SOC to protect the life and performance of the energy storage device. When determining the weight coefficients b, β and γ, first of all, based on the economic and safety priority of the power system operation, set the initial guiding principles. Among them, the cost item is the core optimization objective and should be given the highest weight. The voltage deviation term is directly related to the safe and stable operation of the system, and its weight should be able to ensure that there is a sufficiently significant penalty for over-limit behavior. The SOC variation penalty term aims to smooth the energy storage action and extend the equipment life. Its weight needs to strike a balance between avoiding frequent charging and discharging and ensuring the regulation capacity. Therefore, in this study, the initial range of b is set at 0.5–1.5, that of β at 10–50, and that of γ at 1–10. Then, the Bayesian optimization method is used to conduct an automatic search within the above range to maximize the long-term cumulative reward of the strategy in the validation environment after the training is completed. Finally, after multiple rounds of iterations, the weights b were determined to be 0.7, β to be 30, and γ to be 5. After establishing the model, the study uses MAPPO to train the expert strategy model in the microgrid environment and obtained the expert strategy through training. Through this strategy, a large number of state-action pairs were collected under different states to form an expert experience data set, whose expression is shown in Equation (14) [28].


$$D_{e}=\overset{N}{\underset{i=1}{\left\{\left(s_{i},a_{i}\right)\right\}}}$$ (14)

In Equation (14), N represents the number of collected samples, $D_{e}$ is the expert experience dataset, and $\left(s_{i},a_{i}\right)$ represents the state-action pairs. These samples contain the optimal decisions made by the expert under various conditions, which provide a reference for the generator network to learn in DB-GAIL. In the DB-GAIL framework, the goal of the generator network is to learn the expert strategy and generate action sequences similar to those of the expert. Its optimization objective function is shown in Equation (15).


$$J\left(\theta \right)=E_{s\sim \;D,a\sim \;\pi \theta \left(a|s\right)}\left[\frac{\pi \theta \left(a|s\right)}{\pi \theta_{old}\left(a|s\right)}A^{\pi \theta_{old}}\left(s,a\right)\right]$$ (15)

In Equation (15), θ represents the parameters of the generator network, $\theta_{old}$ is the parameters from the previous iteration. πθ(a|s) represents the action probabilities, and $A^{\pi \theta_{old}}\left(s,a\right)$ is the advantage function used to measure the advantage of the current action in the current state relative to the average policy. Its calculation is shown in Equation (16).


$$A^{\pi \theta_{old}}\left(s,a\right)=Q^{\pi \theta_{old}}\left(s,a\right)-V^{\pi \theta_{old}}\left(s\right)$$ (16)

**Fig 6:**
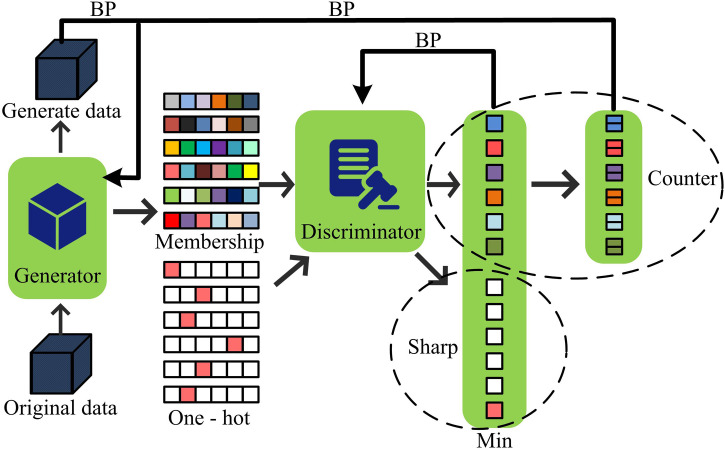
Schematic diagram of the discriminator network.

In Equation (16), Q is the state-action value function, representing the expected cumulative reward after performing an action from a given state, V is the state value function, representing the expected cumulative reward starting from a given state. The discriminator network uses a neural network with a Sigmoid activation function. Its schematic diagram is shown in Fig 6.

As shown in Fig 6, the discriminator workflow in the generative adversarial network. The generated data generated by the generator is input into the discriminator together with the one-hot vector of the non-real label. The discriminator uses a neural network to judge the authenticity of the data. The one-hot vector constrains the generated data features to be more obvious for distinction. The counter on the right counts the number of judgment results to assist in evaluating performance, and the sensitivity measures its data discrimination ability. During training, the discriminator takes the negative of the output result as the loss to back-propagate and update the generator. The two form an adversarial relationship. The parameters are optimized by gradient descent to make the generated data close to the truth and improve the discriminator’s discrimination ability until it is difficult to distinguish between the true and the false and achieve a good classification accuracy. The discriminator network analyzes the input state-action pair and outputs a probability value to judge the possibility that the data comes from the expert strategy. The optimization objective function of the discriminator network is shown in Equation (17) [29].


$$L\left(\phi \right)={max}_{\phi }\;E_{\left(s,a\right)\sim \;D_{e}}\left[log\;D_{\phi }\left(s,a\right)\right]+E_{\left(s,a\right)\sim \;\pi \theta }\left[log\left(1-D_{\phi }\left(s,a\right)\right)\right]$$ (17)

**Fig 7:**
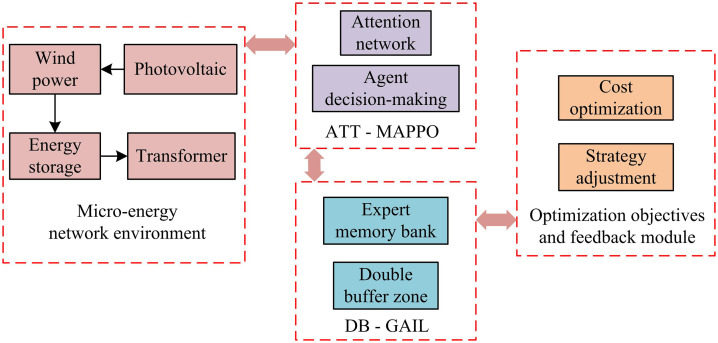
Schematic diagram of collaborative optimization model framework.

In Equation (17), ϕ represents the parameters of the discriminator network, and L(ϕ) represents the optimization objective function value. When the discriminator is unable to distinguish between generated data and expert data, the generator network learns the optimal strategy that is close to the expert’s strategy, achieving collaborative energy optimization for the energy router. The collaborative optimization model framework is shown in Fig 7.

As shown in Fig 7, devices such as wind power, photovoltaic, energy storage, and transformers in the microgrid environment provide the basic energy input for the energy router’s collaborative optimization model. Their output characteristics vary. Wind and solar power exhibit intermittent fluctuations, while energy storage can regulate energy. The ATT-MAPPO first normalizes the data. After processing by a multi-head attention network, the policy neural network makes decisions based on this information. The environment feedbacks information, and the experience is stored for training. DB-GAIL, on the other hand, provides data through the expert memory bank, optimizing the strategy via adversarial training between the generator and the discriminator. The optimization objective and feedback modules aim to minimize operational costs. The improved algorithm adjusts the strategy based on this feedback, creating a closed loop that enhances the microgrid’s energy utilization efficiency and economic benefits. The pseudo code of energy collaborative optimization model is shown in Table 1.

**Table 1:** Energy collaborative optimization model.

Energy collaborative optimization model
Algorithm: ATT-MAPPO + DB-GAIL Training Procedure
# Initialize networks and buffers
Initialize:
Actor networks π_θ for each agent
Critic network V_φ
Discriminator network D_ψ
Expert buffer B_expert
Generator buffer B_gen
for episode = 1 to T do:
# Collect trajectories
s = env.reset()
for t = 1 to H do:
# Get actions from policy (Eq. 4, 15)
a = π_θ(s) + noise for exploration
# Execute actions and observe rewards
s’, r = env.step(a)
# Store transition
Store (s, a, r, s’) in B_gen with priority based on TD-error
s = s’
# Update ATT-MAPPO
for each agent i do:
# Process states with attention mechanism
h_i = Attention(s_i, s_{-i})
# Calculate advantage (Eq. 1, 16)
A = Q_π(s, a) – V_π(s)
# Update policy (Eq. 4, 5, 15)
ratio = π_θ(a|s)/ π_θ_old(a|s)
L_π = min(ratio * A, clip(ratio, 1-ε, 1 + ε) * A)
θ = θ + α_π * ∇L_π
# Update critic (Eq. 2, 3)
L_V = (R – V_φ(s))^2
φ = φ – α_V * ∇L_V
# Update DB-GAIL
# Sample from buffers (20% expert, 80% generator)
batch_expert = sample(B_expert, 0.2*batch_size)
batch_gen = sample(B_gen, 0.8*batch_size)
# Update discriminator (Eq. 6, 17)
L_D = -E[log(D_ψ(s,a))] – E[log(1-D_ψ(s,a))]
ψ = ψ – α_D * ∇L_D
# Update generator with SAC-style optimization
for each (s,a) in batch_gen do:
# Generator reward (Eq. 7)
r_g = log(D_ψ(s,a))
# Update policy to maximize reward
L_G = E[α * log π(a|s) – Q(s,a)]
θ = θ + α_G * ∇L_G
# Update buffers
Update priorities in B_gen
Periodically update B_expert with new expert data
Output: Optimized policy π_θ

## 3. Results

### 3.1. Experimental analysis of the improved algorithm

In order to verify the performance of the improved algorithm combining ATT-MAPPO and DB-GAIL proposed in the study, the study compared it with PPO, GAIL, and PPO-GAIL. The experimental environment is shown in Table 2.

**Table 2:** Experimental environment.

Category	Item	Version model
Hardware environment	CPU	Intel Xeon Platinum 8380
	GPU	NVIDIA A100 80GB
	Memory	64GB DDR4
	Storage	1TB NVMe SSD
Software environment	Operating system	Ubuntu 20.04 LTS
	Programming language	Python 3.8
	Deep learning frameworks	PyTorch 1.13
	Related libraries	NumPy 1.23

**Fig 8:**
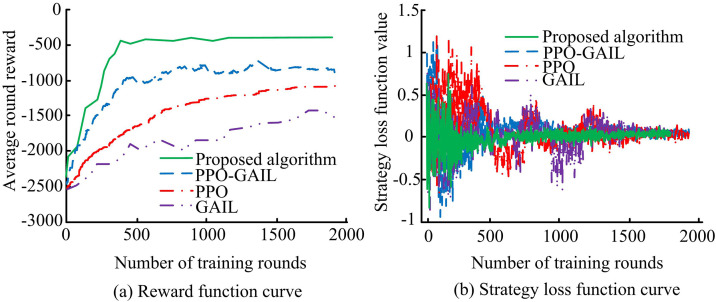
Reward function and strategy loss function curve.

In terms of hardware, the study selected an Intel Xeon Platinum 8380 CPU and an NVIDIA A100 80GB GPU, with 64GB DDR4 memory and 1TB NVMe SSD storage. In terms of software, based on the Ubuntu 20.04 LTS operating system, Python 3.8 was used as the programming language, and the algorithm model was built with the help of the PyTorch 1.13 deep learning framework, supplemented by NumPy 1.23 related libraries for data processing and analysis. The four algorithms were simulated and trained respectively, and their reward functions and strategy loss functions were observed and recorded. The comparison results are shown in Fig 8.

**Fig 9:**
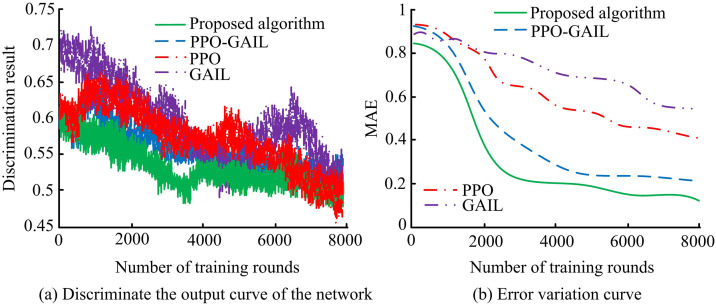
Discriminant network output and error change diagram.

As shown in Fig 8(a), the average round reward of the proposed algorithm increased rapidly after 420 trainings, and finally stabilized at a level close to −410. The rising trend of the PPO-GAIL reward was relatively gentle, but the average round reward also rose to −1000 after 450 times, and finally stabilized at −920 after 1350 times. The PPO reward increased relatively slowly, and the average round reward finally stabilized at about −1200. The GAIL reward increased the least and was the most unstable. As shown in Fig 8(b), in the early stage of training, the policy loss function values of each algorithm fluctuated greatly. As the number of training rounds increased, the policy loss function value of the proposed algorithm decreased first and tended to stabilize after 500 times. The loss function values of PPO-GAIL, PPO, and GAIL also gradually decreased and stabilized, but the degree of fluctuation was relatively large. Experiments showed that the proposed algorithm showed better training effect and stability. To further verify the effectiveness of the proposed algorithm, the discriminant network was used to estimate the probability of the generated strategy to measure the difference in action values. The output results and errors of the discriminant networks of different algorithms are shown in Fig 9.

**Fig 10:**
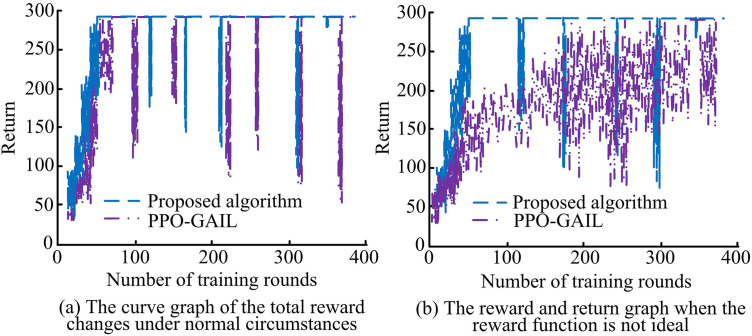
Curve comparison of different reward functions.

As shown in Fig 9(a), as the number of training rounds increased, the output results of the discriminant network of the proposed algorithm decreased rapidly, and it decreased significantly at about 3500 rounds. PPO-GAIL decreased closely afterwards. The decreasing trend of PPO was relatively slow. In the end, the output probability of the discriminant network of each algorithm approached 0.5. As shown in Fig 9(b), as the training progressed, the Mean Absolute Error (MAE) of the proposed algorithm decreased rapidly, and decreased significantly at about 2000 rounds. The MAE value of PPO-GAIL decreased second, while PPO and GAIL decreased relatively slowly. In the end, the MAE value of the proposed algorithm stabilized at a low level, reaching 0.18. PPO-GAIL, PPO, and GAIL also gradually decreased and stabilized, but they were all higher than the proposed algorithm. Overall, the proposed algorithm performed better in improving the discriminant network’s discrimination ability and fitting the generated strategy with the expert strategy, and was able to balance the discriminant network output faster and reduce the error between strategies. In order to verify the robustness of the proposed algorithm, two situations, normal reward and unsatisfactory reward, were set, and the reward return changes of the proposed algorithm and PPO-GAIL were compared. The comparison results are shown in Fig 10.

As shown in Fig 10(a), under normal circumstances, as the number of training rounds increased, the total rewards of the proposed algorithm and PPO-GAIL both showed an upward trend. The proposed algorithm rose faster, tended to stabilize after 50 training rounds, and then only fluctuated 5 times. However, PPO-GAIL rose slightly slower, requiring 80 training rounds to stabilize, and then fluctuated 6 times, with the fluctuation amplitude being slightly larger. As shown in Fig 10(b), when the reward function was not ideal, the proposed algorithm could still maintain a certain upward trend, the return was relatively stable, the number of fluctuations had not changed, and the amplitude had increased slightly. However, the fluctuation of PPO-GAIL had increased significantly, and it had stabilized after 350 rounds, followed by large fluctuations again, and the return stability had deteriorated. This showed that compared with PPO-GAIL, the proposed algorithm converged faster and had better stability under normal reward functions, and had stronger anti-interference ability and better performance. To further verify the practicality of the improved algorithm combining att-mappo and db-gail, the computational complexity of the proposed algorithm, PPO, Gail, PPO-GAIL, and the Deep Deterministic Policy Gradient Algorithm (DDPG-PER) based on priority experience replay were analyzed. All algorithms were run independently 10 times under the same hardware environment. Statistical indicators are expressed as mean ± standard deviation, and the significance of differences was verified using an independent samples t-test. A significance level of 0.05 and *p* < 0.05 indicated that the differences between groups were statistically significant. The results are shown in Table 3.

**Table 3:** Comparison of Computational Complexity of Different Algorithms.

Algorithm	Parameter quantity (ten thousand)	Time complexity	Space complexity	Single iteration time consumption (ms)
The proposed algorithm	18.6	O(n·m·logk)	O(n·m + k)	42.3 ± 2.1
PPO-GAIL	15.2	O(n·m·k)	O(n·m·k)	58.7 ± 3.5
PPO	10.5	O(n·m)	O(n·m)	35.6 ± 1.8
GAIL	12.8	O(n·m·k)	O(n·m·k)	62.1 ± 4.2
DDPG-PER	11.3	O(n·m·log k)	O(n·m + k)	38.9 ± 2.4

Note: n represents the state dimension, m represents the action dimension, and k represents the sample size of the buffer.

As shown in Table 3, the proposed algorithm has 186,000 parameters, slightly higher than PPO, GAIL, and DDPG-PER, but comparable to PPO-GAIL and PER-GAIL. In terms of time complexity, the proposed algorithm is O(n·m·log k_e + n·m·log k_g), significantly lower than the O(n·m·k) or higher complexity of PPO-GAIL, GAIL, and PER-GAIL. In terms of space complexity, the proposed algorithm is O(n·m + k_e + k_g), only higher than PPO and DDPG-PER, but significantly lower than PPO-GAIL, GAIL, and PER-GAIL which use a large mixed buffer. The proposed algorithm takes 42.3 ± 2.1 ms per iteration, slightly higher than PPO and DDPG-PER, but significantly lower than PPO-GAIL, GAIL, and PER-GAIL. Statistical tests show that the difference in single-iteration time between the proposed algorithm and other algorithms is statistically significant (*p* < 0.05). The above results demonstrate that the proposed algorithm, while incorporating advanced mechanisms, keeps its computational complexity within a reasonable range. In particular, it exhibits a significant efficiency advantage compared to other algorithms that combine imitation learning or simply apply PER, proving its practicality in the scenario of energy collaborative optimization for power routers. To accurately evaluate the contributions of the proposed DB mechanism and priority sampling to generative adversarial learning, ablation experiments were conducted. Four comparative models were designed: GAIL, DB-GAIL (single buffer), DB-GAIL (uniform sampling), and full DB-GAIL. GAIL uses a single empirical replay pool with uniform random sampling. DB-GAIL (single buffer) uses a single hybrid empirical replay pool to store all data, eliminating data separation and retaining only priority sampling. DB-GAIL (uniform sampling) retains the dual-buffer structure, but samples are uniformly and randomly drawn from the two buffers at a fixed ratio (2:8), and priority sampling is disabled. All experiments with full DB-GAIL were conducted in the same microgrid simulation environment, using the same ATT-MAPPO as the policy skeleton. The evaluation metrics were expert data utilization, policy update variance, sample efficiency, and final discriminator accuracy. The results are shown in Table 4.

**Table 4:** Ablation Experiment Results.

Model	Expert Data Utilization (%)	Policy Update Variance	Sample Efficiency (Environmental Interaction Steps/10,000)	Final Discriminator Accuracy	References
GAIL	20.1	1.2	35.4	0.022	[6]
DB-GAIL (Single Buffer)	22.8	2.8	28.1	0.048	[23]
DB-GAIL (Uniform Sampling)	49.2	5.1	18.7	0.102	[22]
DB-GAIL	68.5	7.3	12.5	0.156	This study

As shown in Table 4, the expert data utilization rate of the complete DB-GAIL is 68.5%, which is 3.4 times that of the baseline GAIL. This proves that the double-buffered separation structure and priority sampling mechanism can effectively ensure that high-quality expert experience is fully learned. The policy update variance of the complete DB-GAIL is the lowest at 1.2, which is 83.6% lower than that of GAIL. This indicates that by providing high-quality, low-correlation training sample batches, the DB mechanism can greatly reduce the variance of policy gradient estimation, making the learning process smoother and more stable. The number of interaction steps required for the complete DB-GAIL to reach the same performance threshold is only 125,000, which is 64.7% less than that of GAIL. This shows that the higher data utilization rate and more stable updates enable the algorithm to obtain more effective learning signals from each interaction. The final discriminator accuracy error of the complete DB-GAIL is only 0.022, which is much lower than that of other models. This indicates that during adversarial training balance, the discriminator has the most difficulty in distinguishing the behavior of its generated policy from that of the expert policy, which objectively proves the superiority of its imitation effect. In summary, the proposed double buffering mechanism significantly improves GAIL’s performance in terms of training stability, convergence speed, and sample utilization efficiency by optimizing data storage and sampling strategies. To verify the overall robustness of the proposed model for the weight configuration of the reward function, this study analyzed the sensitivity under different weight combinations, and the results are shown in Table 5.

**Table 5:** Sensitivity Analysis.

Weight combination ($\begin{array}{c} b \end{array}$, $\begin{array}{c} \beta \end{array}$, $\begin{array}{c} \gamma \end{array}$)	Proportion of voltage over-limit time (%)	Average daily volatility of energy storage SOC (%)	Comprehensive performance score
1.0, 30, 5	0.12	18.5	1.000
0.7, 25, 3	0.85	22.7	0.972
1.0, 35, 6	0.04	16.3	0.994
1.0, 30, 7	0.11	15.1	0.998
1.2, 28, 4	0.15	19.8	0.997

It can be seen from Table 5 that when the weight combination is (1.0, 30, 5), the proportion of voltage over-limit time of the model is 0.12%, the average daily volatility of the energy storage SOC is 18.5%, and the comprehensive performance score is 1.000. When the weight combination is (0.7, 25, 3), the proportion of voltage over-limit time is 0.85%, which increases significantly. The average daily volatility of energy storage SOC is 22.7%, with increased volatility. The comprehensive performance score is 0.972, maintaining a relatively high level. It indicates that safety and stability can still be maintained even when the voltage and energy storage penalty weights are moderately reduced. When the combined weight is (1.0, 35, 6), the proportion of voltage over-limit time is 0.04%, which is significantly reduced. The average daily volatility of the energy storage SOC also decreases, and the comprehensive performance score is still relatively high. It is indicated that strengthening voltage stability and energy storage protection can ensure system safety and equipment lifespan. When the combined weight is (1.0, 30, 7), the proportion of voltage over-limit time is 0.11%, which remains almost unchanged. The average daily volatility of energy storage SOC is 15.1%, which decreases, and the comprehensive performance score is 0.998. This indicates that further increasing the penalty for SOC fluctuations in energy storage can, while maintaining a safe voltage level, more effectively smooth the operation of energy storage devices and extend their service life. When the combined weight is (1.2, 28, 4), the proportion of voltage over-limit time is 0.15%, the average daily volatility of energy storage SOC is 19.8%, both of which have increased, and the comprehensive performance score is 0.997. It is indicated that after making minor collaborative adjustments to the ownership weight, the model can still maintain excellent comprehensive performance. The above results fully demonstrate that the proposed model has good robustness for the weight configuration of the reward function.

### 3.2. Performance verification of the energy collaborative optimization model

**Fig 11:**
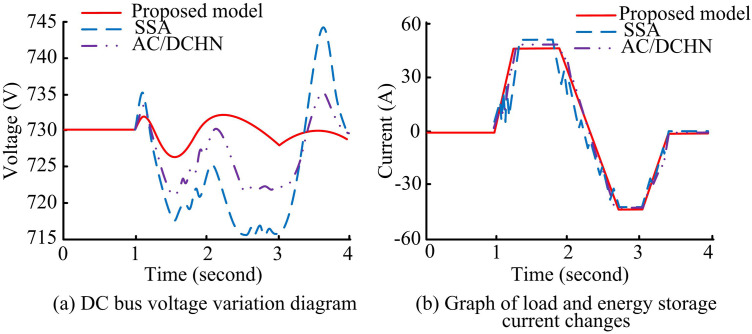
Comparison of DC bus voltage and energy storage current variation over time.

After verifying that the proposed algorithm in the study demonstrated certain advantages, the study conducted a case analysis using the energy collaborative optimization model of the electric energy router, applied to the data from a practical integrated energy system. The analysis was compared with an optimal economic dispatch model of a multi-energy router system optimized by the Sparrow Search Algorithm (SSA) and a coordinated control and dispatch optimization model based on the AC/DC Hybrid Network (AC/DCHN). Considering the needs of the actual integrated energy system data processing comparison experiment, the hardware used 64GB DDR4 memory and 1TB NVMe SSD storage, with the CPU replaced by Intel Xeon E5-2620 v4, and the GPU was NVIDIA Tesla V100. At the software level, the Ubuntu 20.04 LTS operating system and Python 3.8 were used as the programming language, with the PyTorch 1.11 deep learning framework and the NumPy 1.21 library for data processing and analysis. The reason for choosing SSA and AC/DCHN as baseline models is that SSA is a traditional heuristic optimization algorithm widely used in the field of economic dispatching of power systems. It has formed a mature application paradigm in the multi-energy router collaborative dispatching scenario and can directly reflect the performance level of mainstream optimization methods in engineering practice. AC/DCHN is a classic framework for coordinated control of AC/DC hybrid microgrids, which is highly compatible with the multi-energy interactive dispatching scenario of the power router in this study, and can verify the adaptability of the proposed model under the actual power grid topology. To ensure fairness in performance evaluation, all models were built based on the same microgrid parameters and constraints, and the hardware, software, and data processing workflows were completely unified. Furthermore, this study aims to verify the effectiveness of the proposed models in solving specific engineering problems, rather than conducting extensive internal comparisons of DRL algorithms. Direct comparisons with general DRL algorithms such as SAC, TD3, QMIX, and MADDPG would introduce additional complexity due to differences in the matching degree between the algorithm’s design goals (continuous control, discrete cooperation, and full cooperation) and the scenario of this study, making it difficult to fairly measure the model’s engineering practical value in this specific field. All models used the same index system, and experiments were independently repeated three times, with the average value taken, to avoid random errors affecting the objectivity of the results. To verify the advantages of the proposed model in maintaining voltage stability and controlling energy storage current changes in actual operation, the operating data of different models in the actual integrated energy system were compared, and their changes over time are shown in Fig 11.

**Fig 12:**
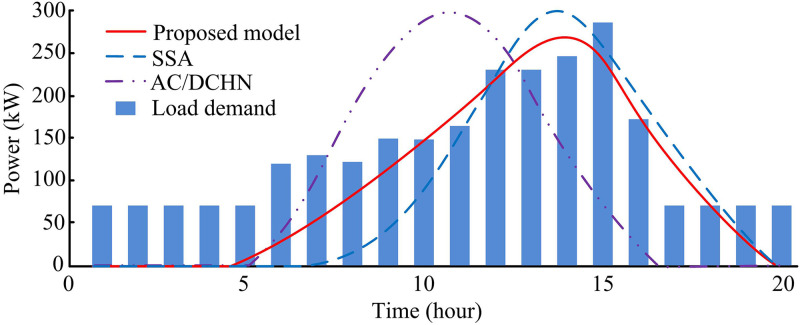
Comparison of photovoltaic power output and load demand.

As shown in Fig 11(a), at the initial moment, the DC bus voltage of the proposed model was 730V, and during the entire process, its voltage fluctuated roughly between 728V and 732V. The SSA model exhibited significant voltage fluctuations, with the voltage rising to 735V around 1 second, then quickly dropping to 718V at 2 second, with the maximum fluctuation range reaching approximately 17V. The AC/DCHN model had relatively small voltage fluctuations, rising to 732V at 1 second and dropping to 722V at 2 second, with a fluctuation range of 10V. As shown in Fig 11(b), the energy storage current of the proposed model increased to 50A at around 1 second, then steadily decreased to -40A at 3 second, with relatively regular changes. The SSA model’s current fluctuated significantly, rising to 55A at around 1 second but experiencing multiple oscillations during the rise, and the decline was also unstable, reaching -30A at 2 second with significant fluctuations. The AC/DCHN model’s current change exhibited fluctuations between the two, rising to 52A at 1 second and falling to -35A at 3 second, with the fluctuation degree being relatively moderate. Overall, the proposed model demonstrated superior performance in maintaining DC bus voltage stability and regular changes in energy storage current. Next, the study conducted optimization experiments on power output and demand for the microgrid using the three models, and the variations in photovoltaic power and load demand under each model are shown in Fig 12.

**Fig 13:**
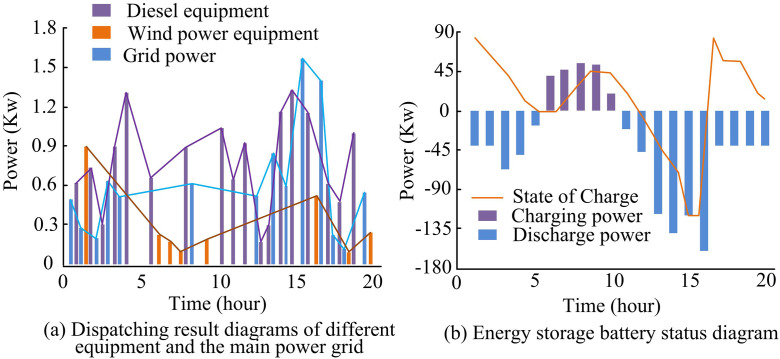
Dispatch results and energy storage battery status.

As shown in Fig 12, the photovoltaic power output under the three models varied in their ability to meet load demand during different periods. From 1–5 hour, the load demand remained at 70kW, then gradually increased, reaching 150kW at 10 hour, and remained between 240-250kW from 12–15 hour, peaking at 280kW at 15 hour before dropping. During the 0–5 hour period, the photovoltaic power output from all three models was far below the load demand, and none could meet the electricity demand. The proposed model was able to meet the load demand well during most of the 10–15 hour period, while the SSA model performed relatively better during the 15–20 hour period. The AC/DCHN model was able to meet the demand during parts of the 10–15 hour period. Overall, the proposed model had a clear advantage in matching photovoltaic output power to load demand, reflecting its superiority in power regulation within the microgrid. When photovoltaic power was insufficient or in excess, other energy supply devices were scheduled, and the output power of wind power, diesel generation, and the main grid, as well as the balance results of the energy storage battery, are shown in Fig 13.

As shown in Fig 13(a), during periods when photovoltaic power was insufficient to meet load demand, such as from 5–10 hour, diesel equipment, wind power, and the main grid actively supplied power. At 1 hour, the output power of diesel, wind power, and the main grid were 0.6kW, 0.9kW, and 0.5kW, respectively. By 5 hour, the diesel equipment’s output had risen to 1.3kW. When photovoltaic power exceeded the required load demand during periods such as 6–10 hour, the output power of these devices was reduced. As shown in Fig 13(b), during the 0–5 hour period when photovoltaic power was insufficient, the energy storage battery was in discharge mode, with a discharge power of 44kW at 1 hour. During the 6–10 hour period when photovoltaic power was excessive, the energy storage battery was in charge mode. Finally, the study conducted an in-depth analysis of the economic indicators for each model, including total operation time and electricity costs for various energy types, with the specific comparative results shown in Table 6.

**Table 6:** Real-time scheduling results comparison of different models.

Model	Total running time (s)	Electricity cost (yuan)	Photovoltaic cost (yuan)	Wind power cost (yuan)	Cost of diesel generator (yuan)	Power grid cost (yuan)
Proposed model	53.32	3846.36	850.23	920.15	1045.34	1030.64
SSA	68.26	4985.96	1020.35	1150.48	1350.67	1464.46
AC/DCHN	90.41	6953.12	1205.48	1320.65	1680.75	2746.24

As shown in Table 6, for total operation time, the proposed model took 53.32 seconds, the SSA model took 68.26 seconds, and the AC/DCHN model took 90.41 seconds, with the proposed model being faster. For electricity costs (in RMB), the proposed model’s cost was 3846.36, the SSA model’s cost was 4985.96, and the AC/DCHN model’s cost was 6953.12, with the proposed model having lower costs. Additionally, in the breakdown of electricity cost components, such as photovoltaic costs, wind power costs, diesel generator costs, and grid costs, the proposed model’s photovoltaic cost (850.23 yuan) and wind power cost (920.15 yuan) were relatively lower than those of the other models, demonstrating its cost control advantages in utilizing various types of energy, which is beneficial for a comprehensive assessment of the performance of each model in integrated energy dispatch. To further simulate the performance of the proposed model in a real-world deployment environment, this study tested it on five platforms: NVIDIA Jetson Orin Nano, NVIDIA Jetson TX2, Huawei Atlas 200 DK, Rockchip RK3566, and Raspberry Pi 4B. The NVIDIA Jetson Orin Nano was configured with a quad-core ARM Cortex-A78AE processor and an integrated GPU, with 4GB of RAM. The NVIDIA Jetson TX2 was configured with a dual-core Denver2 processor and a quad-core A57 processor, with 8GB of RAM. The Huawei Atlas 200 DK was configured with a quad-core Cortex-A55 processor and an Ascend 310 AI processor. The Rockchip RK3566 was configured with a quad-core ARM Cortex-A55 processor. The Raspberry Pi 4B was configured with a quad-core Cortex-A72 processor and 4GB of RAM. 1000 randomly generated state sequences were used as input, and the average inference latency and peak memory usage were recorded. The results are shown in Table 7.

**Table 7:** Inference performance test results of the embedded platform.

Platform	Average Inference Latency (ms)	Peak Memory Usage (MB)
NVIDIA Jetson Orin Nano	10.2	72.1
NVIDIA Jetson TX2	23.5	78.4
Huawei Atlas 200 DK	15.8	165.3
Rockchip RK3566	102.4	88.9
Raspberry Pi 4B	85.6	92.7

As shown in Table 7, regarding real-time inference efficiency, the proposed model has the lowest average inference latency of 10.2ms on the NVIDIA Jetson Orin Nano platform, while the highest is 102.4ms on the Rockchip RK3566 platform. The inference latency on all five platforms is well below 1s, indicating the feasibility of the model completing a single decision within the control cycle. In terms of memory resource usage, the peak memory usage on the five platforms—NVIDIA Jetson Orin Nano, NVIDIA Jetson TX2, Huawei Atlas 200 DK, Rockchip RK3566, and Raspberry Pi 4B—is 72.1MB, 78.4MB, 165.3MB, 88.9MB, and 92.7MB, respectively. This memory usage level is perfectly acceptable for modern embedded systems, indicating that the model can run stably without consuming other system resources. The reason is that the model’s inference response speed is much faster than the conventional control cycle of a power line router, fully meeting the device’s real-time decision-making needs. Furthermore, the memory space occupied by the model during runtime has been lightweight and optimized to fit the memory limit of the embedded hardware, without affecting the normal operation of other functional modules. At the same time, in practical applications, the model only needs to perform forward computation, eliminating the need for repeated training processes, thus placing less computational pressure on the embedded processor and perfectly matching the processing power of the power line router’s embedded CPU.

## 4. Discussion

The study proposed a combination of DB-GAIL and MAPPO based on a double buffer mechanism to build a model to solve the energy collaborative optimization problem of power routers. From the perspective of algorithm performance, the improved algorithm showed outstanding advantages. After 420 training sessions, the average round reward rose rapidly and stabilized at a level of about −410. This data intuitively reflected the good stability and fast convergence ability of the algorithm. Similar to the research results of Jiang’s team using the fusion of PPO and GAIL to achieve agent training and model reality transfer, in the initial stage, the proposed algorithm could converge quickly with the experience of expert strategies, and in the later stage, it could fully explore autonomously in complex environments [30]. In the experiment of the discriminant network estimating the probability of the generated strategy, the MAE value of the proposed algorithm stabilized at a low level, eventually reaching 0.18, and the probability of the discriminant network output approached 0.5 the fastest. Compared with the experimental conclusions of Picinini et al., although the MAE value was slightly higher, the convergence speed of the discriminant network output probability was comparable. The PPO and SAC algorithms proposed by Picinini and other scholars worked together and were combined with the GAIL method, which showed that GAIL could improve the performance of PPO in specific scenarios [31]. This was consistent with the results of the study.

In actual application scenarios, the proposed model showed excellent performance advantages. In terms of maintaining the stability of the DC bus voltage and the regular changes of the energy storage current, its DC bus voltage fluctuation was effectively controlled between 728-732V, which was smaller and more stable than the comparison model. This result was consistent with the battery system experimental results conducted by Meng’s team in 2023. This study constructed a control framework with PPO as the core, and used the gradient descent method to achieve online stable strategy updates [32]. In terms of energy storage current regulation, the reaction speed of the proposed model was delayed by only 0.1 second, and its advantage was more obvious than the method proposed by Meng’s team. In terms of photovoltaic power matching load demand, it could meet the load demand well for 14 hours during the 20-hour operation. Whether it was a period of low or high load demand, the proposed model could flexibly adjust the photovoltaic power output, showing good adaptability and ensuring efficient use of energy. From the perspective of economic indicators, the advantages of the proposed model were more prominent. Its total running time was only 53.32 seconds, and the electricity cost was 3846.36 yuan, which were significantly lower than the other two comparison models. In response to the challenges of privacy protection, energy and load uncertainty, and time-varying scenarios in setting retail electricity prices for renewable energy multi-microgrid systems, the Xiong team proposed a two-level pricing framework based on interval prediction and model-free reinforcement learning. Results showed that this framework minimized power generation costs and network losses, which was equivalent to the effect of the model proposed in the study [33–34].

The contributions of the research were mainly reflected in the following aspects: First, algorithm innovation. The proposed DB-GAIL and MAPPO improved the shortcomings of traditional algorithms in data utilization efficiency, training stability, and multi-agent collaboration, providing a more effective tool for solving the energy management problem of power routers. Second, model optimization. The constructed energy collaborative optimization model integrated the advantages of multiple algorithms, could more accurately cope with complex power environments, and improved the overall level of energy collaborative optimization. Third, practical verification. The effectiveness of the model was fully verified through experiments, providing a practical new solution for energy management of power routers, and strongly promoting the development of this field.

## 5. Conclusion

In the context of the increasing proportion of distributed energy in the power sector, energy routers play a significant role in improving energy utilization efficiency. The study proposed a combination of DB-GAIL and MAPPO to construct an energy collaborative optimization model. The experiments showed that the improved algorithm demonstrated outstanding advantages, with strong reward acquisition capability, faster stabilization of the policy loss function, superior discriminative ability of the discriminant network, high fitting degree between the generated strategy and expert strategies, and good robustness. In practical applications, the proposed model performed better in stabilizing DC bus voltage and matching photovoltaic power with load demand. It also had shorter total running time and lower electricity costs. In summary, DB-GAIL and MAPPO effectively improved the energy collaborative optimization level of power routers, providing a feasible solution for addressing their energy management challenges, and strongly promoting the development of this field.

### 5.1. Limitations and future work

This study also has certain limitations. In real scenarios, power routers are mostly equipped with embedded processors and limited memory, with computing power being only one-tenth to one-fifth of that in experimental environments, which may lead to a decline in the real-time performance of improved algorithms. The impact of hardware failures on training stability was not taken into account in the experiment, while the actual system needs to deal with such unexpected problems. Meanwhile, the computational complexity of the algorithm may become a challenge in large-scale applications. Therefore, in practical applications, it is recommended to set up edge servers at substations or distribution network gateways to undertake part of the training tasks, reduce the local computing pressure on power routers, and control decision-making delays. Model pruning is adopted to compress the model size and adapt to embedded hardware. And take into account more practical factors, such as regional energy structure differences and dynamic changes in the electricity market, to further enhance the practicality of the model. In addition, in the future, efficient computing methods or hardware acceleration technologies will be explored to enhance the operational efficiency of algorithms, and experiments will be conducted in real power grid environments to address the issues of scale expansion and robustness under extreme working conditions.

## Supporting information

S1 File Minimal Data Set Definition. (DOC)
