# Supplementary material for: Energy collaborative optimization of power routing based on PPO and generative adversarial imitation learning
Source: PLoS One. 2026 Apr 8;21(4):e0346372. doi: 10.1371/journal.pone.0346372 (PMC13061251; doi:10.1371/journal.pone.0346372)
Supplement: S1 File — (DOC) [file pone.0346372.s001.doc]

Figure 8. Reward function and strategy loss function curve (Reward function curve)

| Number of training rounds | | 0 | 500 | 1000 | 1500 | 2000 |
| --- | --- | --- | --- | --- | --- | --- |
| Average round reward | Proposed algorithm | -2359.75 | -412.51 | -408.28 | -411.49 | -410.96 |
|  | PPO-GAIL | -2457.32 | -1019.83 | -932.33 | -920.43 | -925.60 |
|  | PPO | -2512.19 | -1647.88 | -1259.13 | -1138.73 | -1079.1 |
|  | GAIL | -2541.75 | -1967.41 | -1851.32 | -1600.81 | -1509.16 |

Figure 8. Reward function and strategy loss function curve (Strategy loss function curve)

| Number of training rounds | | 0 | 500 | 1000 | 1500 | 2000 |
| --- | --- | --- | --- | --- | --- | --- |
| Strategy loss function value | Proposed algorithm | -0.811 | -0.100 | -0.106 | -0.034 | -0.043 |
|  | PPO-GAIL | 0.725 | 0.063 | 0.042 | 0.009 | 0.028 |
|  | PPO | -0.114 | 0.011 | 0.067 | -0.021 | 0.039 |
|  | GAIL | 0.216 | 0.149 | -0.341 | 0.156 | 0.033 |

Figure 9. Discriminant network output and error change diagram (Discriminate the output curve of the network)

| Number of training rounds | | 0 | 2000 | 4000 | 6000 | 8000 |
| --- | --- | --- | --- | --- | --- | --- |
| Discrimination result | Proposed algorithm | 0.587 | 0.549 | 0.521 | 0.510 | 0.492 |
|  | PPO-GAIL | 0.591 | 0.579 | 0.548 | 0.527 | 0.511 |
|  | PPO | 0.613 | 0.620 | 0.574 | 0.543 | 0.504 |
|  | GAIL | 0.694 | 0.637 | 0.558 | 0.535 | 0.534 |

Figure 9. Discriminant network output and error change diagram (Error variation curve)

| Number of training rounds | | 0 | 2000 | 4000 | 6000 | 8000 |
| --- | --- | --- | --- | --- | --- | --- |
| Discrimination result | Proposed algorithm | 0.845 | 0.321 | 0.205 | 0.187 | 0.183 |
|  | PPO-GAIL | 0.926 | 0.559 | 0.292 | 0.236 | 0.211 |
|  | PPO | 0.931 | 0.797 | 0.572 | 0.469 | 0.398 |
|  | GAIL | 0.885 | 0.822 | 0.723 | 0.627 | 0.545 |

Figure 10. Curve comparison of different reward functions (The curve graph of the total reward changes under normal circumstances)

| Number of training rounds | | 0 | 100 | 200 | 300 | 400 |
| --- | --- | --- | --- | --- | --- | --- |
| Return | Proposed algorithm | 0 | 290.42 | 291.18 | 293.16 | 291.46 |
|  | PPO-GAIL | 0 | 285.54 | 289.94 | 284.04 | 287.82 |

Figure 10. Curve comparison of different reward functions (The reward and return graph when the reward function is not ideal)

| Number of training rounds | | 0 | 100 | 200 | 300 | 400 |
| --- | --- | --- | --- | --- | --- | --- |
| Return | Proposed algorithm | 0 | 290.87 | 291.48 | 291.49 | 291.70 |
|  | PPO-GAIL | 0 | 161.87 | 177.08 | 202.03 | 279.07 |

Figure 11. Comparison of DC bus voltage and energy storage current variation over time (Voltage)

| Number of training rounds | | 0 | 1 | 2 | 3 | 4 |
| --- | --- | --- | --- | --- | --- | --- |
| Voltage (V) | Proposed algorithm | 730.16 | 730.16 | 730.84 | 727.93 | 728.82 |
|  | SSA | 730.16 | 735.16 | 718.58 | 715.81 | 729.97 |
|  | AC/DCHN | 730.16 | 732.03 | 722.39 | 722.18 | 729.63 |

Figure 11. Comparison of DC bus voltage and energy storage current variation over time (Current)

| Number of training rounds | | 0 | 1 | 2 | 3 | 4 |
| --- | --- | --- | --- | --- | --- | --- |
| Current (A) | Proposed algorithm | 0.00 | 50.31 | 50.23 | -40.15 | 0.00 |
|  | SSA | 0.00 | 55.24 | 55.33 | -30.23 | 0.00 |
|  | AC/DCHN | 0.00 | 52.17 | 52.30 | -35.36 | 0.00 |

Figure 12. Comparison of photovoltaic power output and load demand

| Number of training rounds | | 0 | 5 | 10 | 15 | 20 |
| --- | --- | --- | --- | --- | --- | --- |
| Power (kW) | Proposed algorithm | 0.00 | 8.35 | 148.39 | 240.32 | 0.00 |
|  | SSA | 0.00 | 0.00 | 89.94 | 258.24 | 0.00 |
|  | AC/DCHN | 0.00 | 0.00 | 292.29 | 74.82 | 0.00 |
| Load demand | | / | 70.66 | 148.39 | 280.86 | 70..66 |

Figure 13. Dispatch results and energy storage battery status (Dispatching result diagrams of different equipment and the main power grid)

| Number of training rounds | | 0 | 5 | 10 | 15 | 20 |
| --- | --- | --- | --- | --- | --- | --- |
| Power (Kw) | Diesel equipment | 0.623 | 1.309 | 1.040 | 1.224 | 1.003 |
|  | Wind power equipment | 0.903 | 0.236 | 0.203 | 0.527 | 0.251 |
|  | Grid power | 0.505 | 0.527 | 0.623 | 1.560 | 0.542 |

Figure 13. Dispatch results and energy storage battery status (Energy storage battery status diagram)

| Number of training rounds | | 0 | 5 | 10 | 15 | 20 |
| --- | --- | --- | --- | --- | --- | --- |
| Power (Kw) | State of Charge | 83.88 | 8.33 | 17.78 | -118.89 | 12.77 |
|  | Charging power | / | -15.56 | -19.44 | -117.78 | -39.44 |
|  | Discharge power | / | 0.00 | 19.44 | 0.00 | 0.00 |
